# Applied public health research - falling through the cracks?

**DOI:** 10.1186/1471-2458-9-362

**Published:** 2009-09-25

**Authors:** Rebecca K Simmons, David Ogilvie, Simon J Griffin, Lincoln A Sargeant

**Affiliations:** 1 MRC Epidemiology Unit, Institute of Metabolic Science, Box 285, Addenbrooke's Hospital, Hills Road, Cambridge, CB2 0QQ, UK

## Abstract

**Background:**

There is a degree of dissonance between the types of evaluative research required by organisations providing or commissioning health care, those recommended by organisations developing evidence-based guidance, and those which research funding bodies are prepared to support.

**Methods:**

We present a case study of efforts to establish a pragmatic but robust evaluation of local exercise referral schemes. We considered the epidemiological, ethical and practical advantages and disadvantages of a number of study designs and applied for research funding based on an uncontrolled design, outlining the difficulties of carrying out a randomised controlled trial to evaluate an existing service.

**Results:**

Our proposal was praised for its relevance and clear patient outcomes, but the application was twice rejected because both funders and reviewers insisted on a randomised controlled trial design, which we had found to be impractical, unacceptable to service users and potentially unethical.

**Conclusion:**

The case study highlights continuing challenges for applied public health research in the current funding climate.

## Background

Primary Care Trusts (PCTs) must demonstrate that the health services they purchase on behalf of their populations are effective and cost-effective [1]. One source of guidance in this area is the National Institute for Health and Clinical Excellence (NICE), which issues evidence-based guidance on promoting good health and preventing and treating ill-health in England. NICE supports the commissioning of evidence-based care by issuing national guidance based on the best available evidence of effectiveness and cost-effectiveness [2]. The production and implementation of NICE guidance reflects an increasing recognition of the importance of an evaluative culture in the NHS [3] which has also resulted in several well-established services coming under increasing evaluative scrutiny. However, there is a degree of dissonance between the types of evaluative research required by organisations providing or commissioning health care, those recommended by organisations developing evidence-based guidance, and those which research funding bodies are prepared to support.

To take one example, exercise referral schemes -- intended to help prevent and treat conditions associated with sedentary lifestyles, such as obesity -- have been widely adopted in the UK. Sowden and Raine note that '...these schemes were encouraged to expand by the Department of Health (DH) before DH-funded evaluations had reported their findings and with little reference to NICE recommendations' [4]. However, there are few published or ongoing evaluation studies of such schemes and a recent Cochrane review found that the quality of randomised trials in this area was generally poor [5]. The limitations identified included high attrition rates, short follow-up periods, a lack of objective measurement of physical activity and limited objective data on health outcomes. On the basis of the available evidence, NICE issued a recommendation in 2006 that practitioners, policy makers and commissioners should neither endorse nor refer people to exercise referral schemes to promote physical activity unless those schemes were 'part of a properly designed and controlled research study to determine effectiveness' [6,7].

Anecdotal evidence suggests that this guidance was interpreted in some PCTs as advice to disinvest in all exercise referral schemes which were not part of such a study. In 2007 the DH therefore issued a clarification, emphasising that PCTs should continue to provide high quality exercise referral schemes for the medical management of conditions such as type 2 diabetes, obesity and osteoporosis, while acknowledging that further research was required to determine the impact of exercise referral schemes on health inequalities and their effectiveness in increasing physical activity levels in adult populations [8].

A service-academic partnership in Cambridgeshire was charged with developing a pragmatic but robust evaluation of existing local exercise referral schemes to improve targeting of the service and guide NHS spending priorities in light of the NICE guidance and a climate of financial constraint. The aim was not simply to evaluate effectiveness, but also to assess whether schemes were delivering as expected and how their impact could be enhanced, for example by improving targeting, uptake and other aspects of implementation. This dual aim reflected an aspiration that a service-academic partnership in public health should be capable of carrying out evaluative research that can both contribute to the global evidence base for the effectiveness of interventions and serve the evidential needs of commissioners of local services. In this case study we report and reflect on the challenges of attempting to design, execute and fund the type of study which appeared to have been called for and discuss the implications for researchers, research funders, service commissioners and the producers of evidence-based guidance.

## Case study

We aimed to assess whether exercise referral was associated with improvements in participants' self-reported and objectively-measured physical activity and anthropometric, physiological and other measures of health status after completion of the scheme (12 weeks) and in the longer term (one year), thereby addressing many of the identified limitations of previous research [5]. Leisure centre staff were trained in anthropometric, physical activity and fitness measurement and standardised equipment and operating procedures were used to ensure valid and reliable data collection. We also aimed to understand how effective the service was in practice, and how its targeting, uptake and implementation could be improved, by assessing who was being referred, the reasons for referral, completion rates, and the socio-demographic and other characteristics of completers, non-completers and those who derived most benefit.

Mindful of NICE's stipulation of a 'properly designed and controlled' study [6], we considered the trade-offs between a variety of 'ideal' and pragmatic study designs [9] and between the competing merits of internal validity (minimising bias and confounding within the study) and external validity (producing findings applicable to the local context and the population at large). A randomised controlled trial (RCT) would have ensured that intervention and control groups were comparable on baseline characteristics, and process measures could have been collected as part of such a trial. However, we were concerned about the ethical implications of removing an established service and restarting it as a trial, as well as enrolling participants in a study in which they would have had to accept a 50% chance of not joining an existing exercise referral scheme, given that they had recently been identified as in need of a tailored exercise programme. Consultations with exercise referral staff and service users confirmed that even if this design had involved randomisation to a waiting list rather than a no-intervention control group, this would have been unacceptable to participants, resulting in low recruitment and impaired retention in the control group with the associated risk of bias and limited external validity. Indeed, the difficulties in recruiting and maintaining a control group randomised to a waiting list are well described by Isaacs *et al *in their single-centre RCT of an exercise referral programme in Barnet, outer London [10].

A non-randomised controlled study would have addressed the ethical concerns associated with randomisation. A control group with a similar socio-demographic and health profile to that of the intervention group would have been recruited from the local community. The control group would have undergone the same measurements and follow-up as the intervention group but would not have received the tailored exercise programme. However, patients referred to exercise referral schemes do not constitute a homogeneous group which can readily be 'matched' in aggregate, but reflect a diverse range of medical histories and socio-demographic backgrounds, and even if a comparable control group could have been constructed according to these parameters, it is likely that people who take part in such a scheme differ in important ways from those with similar medical and socio-demographic characteristics who do not engage with this type of service [11]. In addition, there would have been little incentive for individuals to enter the control arm of such a study if they had no chance of receiving the service or if they were to be added to a long waiting list. Identifying and recruiting a suitable comparison group would therefore have been difficult and might have introduced selection and response bias.

We therefore selected an uncontrolled before-and-after cohort design as the most feasible and ethically acceptable study design in the situation. Although this design would have had lower internal validity, making it more difficult to attribute any observed changes in health status to exercise referral alone, it would have had strong external validity in terms of assessing the targeting, uptake, implementation and impact of local schemes. We secured ethical approval for this study design and applied for local National Institute for Health Research (NIHR) funding for the study, outlining the difficulties of carrying out an RCT to evaluate an existing NHS service. Our proposal was praised for its relevance and clear patient outcomes, but both the funding body and the external referees insisted on a randomised controlled trial design comparing exercise referral with no exercise referral and the proposal was twice rejected. Since none of the local partner organisations was able to fund the study, it was abandoned following successful completion of a pilot study.

## Discussion

In trying to establish this study, we faced both service challenges (promoting a research culture among exercise referral staff, and engaging GPs and practice managers) and academic challenges (selecting an appropriate design and securing funding). It was potentially difficult, for example, to work as part of a team evaluating a service when the employment of some of the wider stakeholders was potentially dependent on the continuation of the service. Furthermore, while feedback from the funding body praised our proposal as being relevant with clear patient outcomes, the referees also insisted on a randomised controlled design, which we had found on consideration to be impractical and unacceptable to service users, and potentially compromised by limitations to internal and external validity. We were also concerned with the ethics of deliberately withholding an established NHS service. Sowden and Raine [4] concur with our findings by arguing that the experimental evaluation of exercise referral in England is now unlikely because of the widespread assumption of effectiveness, the comprehensive coverage of the schemes, the indirect adverse consequences of dismantling the schemes, and the lack of appropriate process and outcome data.

Our experience thus suggests a degree of dissonance between the types of research required by the NHS, those recommended by NICE, and those which research funding bodies are prepared to support. This reflects a central tension in intervention research in public health between the need to establish evidence of the efficacy of an intervention under ideal conditions and the need to understand whether a potentially efficacious intervention is actually effective in practice and how its implementation can be optimised. Research funding bodies have traditionally supported the former, 'purer' type of research rather than the evaluation of services as 'real world' interventions [12].

### Which study designs are appropriate for applied public health research?

Some funders or peer reviewers may start from the assumption that an RCT is what is required, instead of starting by asking 'What is the research question?' followed by 'What is the most appropriate study design to answer that question in this context?' RCTs are rightly regarded as the gold standard for evaluating efficacy, but their utility for addressing questions in public health intervention research is not universally or uncritically accepted [13]. Macintyre and Petticrew describe a common misconception '...that it is adequate to know that some intervention does good in general, and that it is not necessary to know how much good, at what cost, via what mechanisms, or for which subgroups of the population'[14]. Answers to these latter questions may be particularly important to policy makers and commissioners, for whom evidence-based public health must rely on a variety of types of evidence, including that from qualitative and observational studies [4,15,16]. In addition, RCTs usually start from an assumption of equipoise -- i.e. a position of not knowing which of two competing interventions is more effective, or not knowing whether an intervention is likely to be beneficial or harmful [17] -- but this position is not clear with regards to exercise referral schemes. For instance, in a recent systematic review based on 18 studies (including six RCTs), exercise referral was shown to have a small but significant effect on increasing physical activity in some people [18]. While this suggests that equipoise may no longer be an issue, the small effect size and poor quality of many of the studies included in the review, as well as the lack of data on cost-effectiveness, indicates that the situation is not straightforward. Conversely, given that there is evidence for benefit in some people, there may not be enough equipoise to remove an established exercise referral service and restart it as a trial. The authors recommended that more research was needed to understand uptake and adherence, and also suggested the use of well-conducted qualitative studies. Furthermore, although physical activity has been consistently shown to be imprecisely measured by self-report [19], none of the RCTs in the systematic review incorporated objective measurement, a limitation which our proposed study design would have overcome.

The limitations of observational studies of exercise referral schemes are well known, including moderate participation rates, lack of long-term follow-up and poor compliance [18]. However, methodologically weaker RCTs (for example, those with small numbers of participants, low and differential retention rates, imprecise outcome measures and lack of attention to allocation concealment) should not necessarily "trump" methodologically stronger observational studies [20]. Many RCTs have highly selective inclusion criteria, and even among the eligible population have low recruitment rates (if indeed they are even reported), leading to unrepresentative study samples. In the context of exercise referral schemes, people who agree to be randomised to the possibility of not receiving the service, or who agree to allow their GP to refer them to such a trial, are likely to be systematically different from the population who might participate in this type of intervention outside the setting of an RCT, and the service settings in which an RCT is permitted to take place are likely to be systematically different from those in which the service providers decline to permit an RCT. There is clearly a spectrum from efficacy to effectiveness, and an apparent trade-off between internal and external validity. As such, the choice of study design should not be considered the sole criterion of quality. Indeed, the quality of individual studies is now receiving greater emphasis in the formulation of evidence-based guidance than was originally the case [21].

In general, there is a need to broaden the scope of the criteria that are used to appraise and evaluate public health interventions, including the use of qualitative and observational data [4,15,16]. In some situations, as with our proposed evaluation of an existing service, an RCT maybe neither an ethical nor a pragmatic choice, nor does it necessarily provide the most relevant information or the most unbiased estimates of effects. Evidence-based public health must therefore rely on a variety of types of evidence, often in combination. As Victora *et al *state "...randomisation, without further analyses for adequacy and plausibility, is never sufficient to support public health decision-making, regardless of the level of statistical significance achieved" [13].

### Who should specify and fund applied public health research?

In the wake of the Cooksey report on the funding of health research [22] and the formation of the Office for Strategic Coordination of Health Research (OSCHR), there is some confusion as to what constitutes public health research in the UK and who should be funding it. The new NIHR public health research programme goes some way towards addressing the need for more applied intervention studies outside the NHS, but much public health research appears at risk of falling through the cracks between the slabs of 'basic' research (the responsibility of the Medical Research Council) and 'applied' and clinical research (the responsibility of NIHR), or of being assessed against criteria more applicable to 'basic' research, in which the main currency of success tends to be the number and 'quality' of research publications. Successful translational or applied public health research is more appropriately characterised by evidence of a subsequent change in practice or service provision and translation into population health gain -- outcomes which are likely to depend on greater communication and collaboration between academic partners and those defining service needs to make research projects a success. In light of our experience, we would therefore advocate opportunities for greater dialogue between applicants for funding and those reviewing, awarding and advocating research funding (except where applications have no merit at all). This would promote a more thoughtful consideration of the feasibility of what the referees are suggesting and the development of study designs that are acceptable to research participants, users, and funders.

Opportunities for greater dialogue should also extend to producers of evidence-based guidance such as NICE. There is no mechanism for ensuring that NICE research recommendations are acted upon, and while not all funders will share NICE's research priorities, perhaps decisions by NHS funding bodies should be more closely informed by them. Bidding for funds is always competitive and many factors contribute to a decision to rate some proposals more highly than others. Producers of guidance, and commissioners and practitioners receiving guidance should have due regard for the quality and quantity of the evidence underpinning recommendations before acting on them, since guidance development groups working on public health topics are often in the difficult position of having to make recommendations based on limited evidence. It may also be helpful for bodies issuing evidence-based guidance, such as NICE, to specify acceptable study designs in their recommendations for further research, make clear the conditions under which interventions should or should not be implemented, and explain what health care providers should do while the research called for is being completed. In this context, where a recommendation is made not to offer an intervention outside the setting of a controlled research study because evidence for its effectiveness is described as 'equivocal', it should be made clear whether the equivocation is between a beneficial effect and a harmful effect (in which case the condition of equipoise may be satisfied) or between a beneficial effect and no effect (in which case the condition of equipoise may not be satisfied and commissioners may reasonably decide, in the absence of a more effective alternative course of action, to give existing services the benefit of the doubt until the evidence base is populated with stronger evidence either way). Costs should also be considered when describing results as equivocal. If a service or the evaluation of a service is extremely expensive, this may also affect the condition of equipoise.

## Summary

There is a degree of dissonance between the types of evaluative research required by organisations providing or commissioning health care, those recommended by organisations developing evidence-based guidance, and those which research funding bodies are prepared to support. Applied public health research may face an uncertain future until we are clear about the level and type of evidence that is sufficient to inform NICE guidelines and justify the provision of health services, and we have the scale and availability of research funding to meet these requirements.

## List of abbreviations

DH: Department of Health; NHS: National Health Service; NICE: National Institute for Health and Clinical Excellence; NIHR: National Institute for Health Research; PCT: Primary Care Trust; RCT: Randomised controlled trial.

## Competing interests

SJG has been awarded an NIHR programme grant. He sits on MRC and NIHR funding panels, and reviews for several research funding bodies. DO has served on a NICE programme development group.

## Authors' contributions

RKS is a post-doctoral epidemiologist who coordinated the pilot study outlined in this paper. SJG and DO (consultant clinical academics) and LAS (a consultant in public health medicine) are involved in building service-academic partnerships. All the authors contributed to initial ideas and to the serial drafts and read and approved the final manuscript.

## Pre-publication history

The pre-publication history for this paper can be accessed here:


